# The Ambiguous Impact of Performance Measurement on Service Quality

**DOI:** 10.3389/fresc.2022.878338

**Published:** 2022-05-11

**Authors:** Jan Tøssebro, Odd Morten Mjøen, Rebekka Bruteig

**Affiliations:** ^1^NTNU Social Research, Trondheim, Norway; ^2^Department of Social Work, Norwegian University of Science and Technology, Trondheim, Norway; ^3^Department of Mental Health, Norwegian University of Science and Technology, Trondheim, Norway

**Keywords:** performance measurement, community services, disability, management, quality

## Abstract

**Background:**

Performance measurement is growing in importance as a management tool in services for disabled people.

**Aim:**

The aim of this article is to add to the existing literature by exploring (a) the motivation for the introduction of such measurements, (b) the reasoning behind the choice of current indicators, and (c) the impact of performance measurements on service delivery.

**Methods:**

(1) A study of documents (national and, if available, also local) on the motivation for, choice of, and implementation of quality measurements, and (2) interviews with top and middle managers in community services for people with intellectual disabilities or mental health difficulties.

**Results:**

A varied set of motivations have been identified, including the intention to introduce a more facts-based and transparent governance, the need for information that supports the management of scarce resources, and as a tool in the development of service quality for users. The motivation appears to be dependent on level of government, and the attitude among service unit managers tends to be ambivalent; they want performance measurements but cannot see how to measure the important aspects of service quality. The choice of actual indicators is subject to a process bias; that is, one measures what is easily available in administrative systems. The results concerning impact on services are less clear and also context dependent. We have identified usage in the search for cost-cutting possibilities, defense against critique, and that reporting runs the risk of reinforcing routinization of services.

**Discussion:**

The possible impact on services is discussed. Layers of ambiguity are outlined, as measurements can be tools both for quality development and in the defense of current services against “unrealistic demands” from the media or stakeholders. The measurements tend to be used more as sources of governance information than tools for quality development.

**Conclusion:**

The impact of quality measurement is rather ambiguous. On the one hand, it functions as a tool for budget control, whereas on the other hand, unit managers call for better measurement of user outcomes and expect that such measurement can balance the current preoccupation with input indicators, such as expenditures.

## Introduction

Performance and quality measurements are increasingly used for a wide range of purposes in disability services and policies. This includes increasing importance as a management tool—for monitoring, reporting, evaluation, comparison, accreditation, and as input to service development. In this context, one important issue is whether measurements are reliable and relevant to service quality. Studies of national standards for accreditation suggests that this is not always the case (1, 2). A substantial effort is being made to improve these types of measurements, among others, by making them more oriented toward outcomes for service users (3, 4).

The measurement of service quality can, however, also be viewed as a part of a more pervasive trend in the management of health and care services to use a wide set of performance measurements. In this broader perspective, it is important to ask what is measured in practice, how performance measurements are used as management tools, and how this impacts the services and the everyday lives of service users (i.e., the “doing” of performance measurements). This is about intended purposes, but equally important, whether there are unintended consequences. This issue has not been given much attention in research on disability services (5), but it has been explored in more depth in general studies of public governance (6, 7).

The introduction and widespread use of performance measurements can be seen as a “child” of the so-called New Public Management (NPM) (8, 9), including, but not limited to, the marketization that in many cases followed such reforms in governance. The motivation for the introduction of performance indicators within NPM is linked to principles like transparency, accountability, and benchmarking. Transparency means among others that (a) politicians should know whether one gets value for money, (b) that users should be informed before making choices on service provider (whether they have a choice or not), and that information on quality should be publicly available. Accountability concerns the “purchasers” need to evaluate whether providers deliver according to the agreed-upon contract, and the need of management to document the performance of the services they are heading. It is thus part of quality assurance systems. Benchmarking has to do with monitoring developments over time and determining how a service compares with other similar services, as a means of uncovering needs for action. Overall, the measurement of facts is intended to make the whole process less reliant on the discretion or opinions of professionals.

Although the arguments for performance measurements appear, at least partly, both reasonable and timely, it has been launched heavy criticism regarding the practical consequences for public services (6). One has pointed to the risk of goal displacement (7, 10, 11) as a possible consequence of among others:

- *Tunnel vision*: one sees what is measured, and aspects of care that are not measured lose importance— “what is counted is what counts.”
- *Target fixation*: a complex goal structure is reduced to a few easily measurable aspects (10).
- *Strategic behavior*: staff and management adapt their behavior to the indicators rather than to the mission of the service— “indicators replace goals.”
- *Process bias*: one tends to measure what is easily measured. In practice, this easily slips into input factors (such as staff education) or process descriptors (such as completion of duties/tasks) (12), whereas social aspects and quality of care are given less attention (10, 13).

In current practice, the terms quality indicators and performance measurements are sometimes used interchangeably, but sometimes does the former primarily refer to user outcomes, while the latter is wider and includes a number of input (production) factors and process descriptors. These are very different kinds of measurements used for different purposes but are nevertheless part of the same trend in public governance. In Norway, where this study is conducted, the term “quality indicators” is typically used as an overall concept, but with the risk that a possible process bias transforms the concept from user outcomes to the measurement of input and process indicators. In this article, such a possible transformation will be addressed. We therefore use performance measurement in a broad sense as our main concept and look into the employment of many types of indicators, including user outcomes as well as process and input descriptors.

It is an empirical question whether intended aspects or more questionable side-effects will dominate the “doing” of performance measurements. The impact obviously partly depends on the quality, accuracy, and orientation of the indicators in use as well as how it is used as a management tool and the extent to which one is able to minimize the possible impact of pitfalls. Determining the impact of performance measurements is thus an empirical task, but this can hardly produce simple or generalizable answers, as the impact is dependent on the context and the practical use of the indicators. Thus, what we will outline is more like a landscape where one needs to be aware of contradictory and ambiguous processes. The aim of this article is thus to add to the existing literature on quality measurement in disability services by exploring this landscape in one country: Norway. In this study, we aimed to (i) address the intentions or motivation behind the introduction of performance measurements, (ii) describe the indicators currently in use (i.e., the choice of indicators), and (iii) broaden our understanding of the “doing” of performance indicators.

### The Norwegian Setting

This empirical study took place in Norway. Many countries introduced performance measurements in response to the marketization of services (2), but this was slightly different in Norway. Although some private providers exist, their role is negligible in community disability services. The main service provider is the local authorities (municipalities). A purchaser-provider split was nevertheless introduced, partly to prepare for possible marketization and partly to professionalize decisions about levels of support (i.e., to protect decisions against the potential self-interest of people involved in service provision). The introduction of performance measurements was linked in some cases to this purchaser-provider split, but there was also a general shift from a social-policy reasoning focused on living conditions to a reasoning that addressed the role of quality issues in the internal control systems that became mandatory in health and care services in the 1990s (14). In 1995, the Norwegian Board of Health Supervision launched an action plan aimed at the introduction of quality development and management as core components of such internal control systems (15).

Performance measurement did not initially play a vital role in this. Instead, theories on quality development in organizations, with explicit reference to Deming's cycle (16), was influential. This theory is based on ideas such as continuous development, involvement of all parties, reflection, and management engagement. Indicators played a minor role compared to reflection among managers and employees and their actions to improve on identified shortcomings. After 2000, the approach of the national government gradually transitioned away from a “reflective process” and assumed a “measurement” orientation. Peoples' opinions came to be viewed as subjective and should be replaced by facts. In the early phases, the shift was accompanied by a warning: the measurement of hard facts should be a supplement, not a replacement, for professional discretion (17). Some years later, this warning was forgotten, and quality quantification came to the forefront. In 2011, the Directorate of Health was given the task of developing national quality indicators for the entire health and care sector. This includes state-run hospitals and specialized medical services, and a range of services organized by the local authorities, including nursing homes, care for elderly people living at home, general practitioners, child health centers, services for people abusing substances—and community services for disabled people. Thus, community services for disabled people tend to be organized as a part of this large and rather mixed local health and care sector.

The local authorities were in 2011 by law obliged to have a quality assurance system for local/municipal health and care services, but it was—and still is—optional to use the national indicators. Local authorities in Norway have substantial autonomy in how they organize and perform their duties, including whether they use performance measurements and which they adopt. The practical consequence was a rather uneven development between local authorities and across service sectors. The system is much more elaborate, for instance, in education or employment services than in care. Within the health and care sector, it is more developed in short-term treatment services, such as hospitals, while in community care for disabled people, it is less developed. According to a 2014 overview (18), 60% of Norway's more than 400 municipalities, including all the larger municipalities, had introduced performance indicators in their governance system. Among municipalities using such indicators, more than 80% had introduced them into the health and care sector. The study found, however, that there was general agreement among respondents from local authorities that quality measurement was particularly challenging in the community care sector, as there were few available indicators that actually measured quality. Despite reservations, local authorities nevertheless aimed to make performance measurement a core tool in the management of local health and care services.

## Methods

The data employed in this article is from a qualitative study of the “making” and “doing” of quality indicators in parts of the community health and care sector in Norway, including nursing homes, services for people with mental health difficulties, and people with intellectual disabilities. The data used in this article are from the running of group homes/clustered housing and activity centers for people with intellectual disabilities or mental health challenges. Data from nursing homes or personal assistance schemes are not included. The data comprises policy documents, indicator-based quality reports, interviews with two levels of management, focus groups with people working on the implementation of performance indicators, observations at a meeting between developers of performance measurements and stakeholders, and a dialogue conference. The data sources vary with the above-listed sub-objectives of this article (motivation, choice, and “doing”) and is outlined accordingly.

The data on motivation for the introduction of performance measurements differs between levels of the governance system: the national level, the local authority level, and the service unit level. At the national level, data on motivation is primarily extracted from policy documents, such as white papers, circulars, and recommendations from the government or the Directorate of Health. Relevant documents from 1995 to 2020 are studied with a special focus on arguments related to governance principles, quality assurance, and performance measurement. The issue of performance measurement was rarely raised in disability policy documents but primarily in documents addressing the local health and care sector in general, including community care for disabled people.

At the local authority level, few or no documents exist that convey the motivation behind performance measurement. Our primary data source at this level is management interviews. We have conducted interviews with directors (top managers) for the entire local health and care sector in five local authorities, one medium-sized and four large—by Norwegian standards. We also conducted an interview with a former local health and care director that had the reputation of being an innovator in the introduction of performance measurements at the local level. Two focus group interviews were conducted with professional civil servants working on the development and choice of performance indicators as well as interviews with seven service unit managers. All interviews were conducted in 2021.

The service unit managers were at the middle level (the level above first-line managers) and responsible for several community settings (mainly group homes/clustered housing and activity centers) and a staff of 100–500 people. These managers are in a key position regarding performance measurement, as they prepare reports for top management and initiate possible actions to improve quality at the service level. They are accountable for performance and for keeping the budgets. Five of the informants headed services for people with intellectual disabilities, and two services for people with mental health difficulties.

The interviews addressed all the sub-objectives (motivation, choice, and “doing”). The motivation questions addressed why performance measurements were introduced, what they were meant to achieve, and the purpose for which they were used (monitoring, reporting, comparison, etc.,). The interviewees were also asked to share their personal opinions about and experiences with these types of indicators. The data on motivation collected from the interviews were supplemented by points raised during the dialogue conference (see details below).

The data sources on choice of indicators were partly a mapping of actual choices (existing indicators), partly interviews on the reasoning behind choices, and partly issues service units were expected to report on. The mapping included proposed national indicators (Directorate of Health) and indicators used locally. There also exist a collaboration among larger municipalities to establish common indicators in order to facilitate comparisons with each other (benchmarking)—the so-called ASSS collaboration (Aggregerte Styringsdata for Samarbeidende Storkommuner—aggregated performance data for collaborating large municipalities). The indicators used in this collaboration was also included in our mapping. Interviewees were the same individuals as mentioned above. The interviews addressed which indicators were chosen and why, what service units reported on, and reservations they held about the quality and validity of the chosen measurements. We also observed a meeting between developers of performance measurements and representatives of disability associations about the choice of indicators in services for people with intellectual disabilities. The meeting occurred because the city council had asked for regular reports on the quality of services for this group of users and that stakeholders should be involved in the choice of indicators. Representatives from three user associations and three civil servants who work for the local authority participated in this conversation.

The question about the “doing” of performance measurements is primarily addressed at the service level. The main data source for this issue is thus interviews with the same service unit managers that were mentioned above. Interviewees discussed what they saw as the positive and more problematic effects of performance measurements, including what they believed was the future potential of such measurements. The informants were given opportunities to raise issues that they found relevant and to express their opinions. We also organized a one-day dialogue conference on the impact of performance measurement on services (November 2021). Participants represented six local authorities (two large, two medium-sized, and two small), one labor union, one professional organization, three associations for disabled people, and one non-governmental organization (NGO). There were 36 participants in total. The people from local authorities represented top management in the health and care sector (*n* = 2), professional civil servants involved in systems for quality assurance (*n* = 8), service unit managers (*n* = 7), first-line managers (*n* = 7), and representatives for direct care staff (*n* = 2). The conference covered the types of performance measurements currently in use, their perceived benefits, potential problems, and how they can be improved.

One limitation of the current study is a lack of data from street-level staff. To account for this, we have added data to the “doing” section that comes from an earlier study that took place in group homes for disabled people (2017). We employ data from three focus groups with 14 experienced staff members working in group homes (19).

The interview data was analyzed as follows: Interviews were recorded and transcribed verbatim, and the software package NVivo version 12 was used to support data management and retrieval. The authors began by carefully reading the transcripts to gain an understanding of the content. Transcripts were analyzed with an inductive approach through thematic content analyses to identify common patterns and themes (20). All authors examined and revised the themes and responses related to the sub-objectives (motivation, choice, and “doing”). Interview data were compared, clustered, and placed in preliminary themes. This process continued iteratively until a set of themes, each containing sub-themes that captured the range of experiences and views, were identified.

## Results

### Motivation

#### National Level—Facts-Based and Transparent Governance

Initially, in the 1990s, the motivation for creating quality assurance systems was rather implicit. The objective was simply to increase awareness and pay more systematic attention to quality and systems for quality assurance (15, 21). The arguments for more systematic use of performance measurements emerged gradually and was linked in the beginning to the need for measurable standards if the local authority decided to purchase services from private providers. This was followed by the recommendation that the municipal council should establish standards on sufficient quality of services, purchased or not, that could be used as a benchmark in the evaluation of current services (17).

More elaborate arguments for quality/performance measurements were introduced in the 2011 law on local health and care services (22) and developed in a follow-up white paper (23). The arguments were highly influenced by New Public Management and can be summarized as follows:

- *Facts based governance*: There is a need to base evaluations, monitoring, and reports on facts. The role of opinions and discretion should be reduced, not necessarily in the day-to-day delivery of services, but for purposes of governance.
- *Transparency*: Policymakers (the municipal council) should be able to monitor whether one gets value for money, whether services are of a sufficient quality, and that the level of non-conformance is acceptable. Furthermore, users and the public should be informed about the quality of the services.
- *Benchmarking*: When monitoring whether services perform well, it is necessary to have a benchmark. The standard for comparison could be explicit local standards, changes over time, other service units under the same local authority, or services in other jurisdictions.
- *Governance*: The performance measurements should be employed as input to the local system of governance. This applies to a) reporting, b) input to performance reviews, such as regular review meetings between top management and unit managers, and c) be the basis for internal evaluation and plans for quality development.
- *User focus*: Although the focus is on the governance system, sensitivity to user needs is addressed in all policy documents, and in the context of performance measurement, this is transformed into a recommendation to employ user surveys.

These points relate to the explicit discussion of quality indicators, but because the need for data related to governance purposes is linked to the introduction of quality measurement, some duality exists. Thus, there was also introduced statistical reporting to the national government on number of users, services and expenditures in local health and care. This information is meant for national planning and governance purposes and discussed as part of the performance measurement system (24) but is not subject to the above guidelines.

#### Local Authority Level—Management of Resources

The attention paid to performance measurements varies considerably between local authorities depending on the population size of the municipality. Larger municipalities tend to view performance measurements as important in the development of the health and care sector, for monitoring, planning, and funding of services. Smaller municipalities find the situation more transparent and feel less of a need for quantification: “I do not see the need for such indicators. The service users are our neighbors” (unit manager).

For larger municipalities, the need for indicators is perceived in the context of governance of the service sector and efficient management of resources. There is a need to monitor the current state of services, assess use of resources, and evaluate to what extent one gets value for money. This is partly related to transparency. Numbers are seen as an efficient way of creating an overview and communicating information, both internally amongst administration and externally to the municipal council and the public:

*It would be good if we could illustrate as much as possible with numbers. This is about communicating to the municipal council. To explain issues based on a few tables is easier and makes it easier to understand*. (Unit manager)

However, issues related to benchmarking or comparison appear to be more important. The larger municipalities participate in the ASSS-collaboration and thus cooperate when it comes to performance measurement. The use of common indicators is intended to provide an opportunity to learn from each other, as the municipality can analyze areas where it, for instance, spends more (or less) than comparable municipalities. This is seen in the context of self-assessment (“how do we perform?”) and applies to expenditures, use of resources, and, in principle, the outcome or quality for users. However, measuring quality for users is viewed as complicated and, in general, one misses better quality-relevant measurements.

A few directors in the local health and care sector and one unit manager were explicit that performance measurement could also be a tool in the management of expectations. They argued that an important challenge is (what they perceive as) increasing expectations of families, which exceed what is possible to deliver within the current resource situation. The municipalities face criticisms about the level and quality of services from media, politicians, families, and user organizations. The management believes that performance measurement could be helpful in sorting out “fair” from “unfair” criticisms, for instance by showing statistics on consumer levels of satisfaction or levels of expenditures compared to other municipalities.

Quality improvement was also an issue, but it was less striking in the interviews with top management when it came to use of indicators. One should, however, note that the majority of the top manager interviewees expressed reservations about performance measurements due to the lack of or dubious relevance of user outcome measures. The input and process indicators were generally considered to be of sufficient quality, but some expressed concerns about the reliability of comparisons between municipalities.

In summary, the top management of local authorities pointed to quality development, transparency, and the need to assess performance, but issues related to the efficient management of resources and the governance of the sector were at the forefront of their motivation.

#### Service Level—Tools for Quality Development of Services

At the service level, unit managers are ambivalent to performance measurement. On the one hand, they ask for more systematic use of such indicators, but on the other hand, they doubt that the complexity of the service can be adequately represented by a limited set of indicators:

*It is difficult to develop good indicators of quality. I think it would be good to have more indicators. We should measure more*. (Unit manager)

*One cannot really measure quality, only whether a task is done and documented or not*. (Unit manager)

The unit managers' arguments for performance indicators addressed the need to monitor service quality and access more hard facts, better tools for the identification of areas in need of improvement, data on changes over time, and input to quality improvement. Unit managers also argued that more measurement of user outcomes could facilitate a shift in focus in their annual reviews with top management because such indicators could strengthen the focus on topics other than budget issues. They see user outcome measurement as a tool that can be used to increase the focus on service quality. Like the top managers, some of the unit managers recognize the communication advantages of numbers (i.e., communication to the municipal council, top management, and user representatives). They also believe that performance measurement would be a useful communication tool in service development talks with staff. Furthermore, they look for opportunities to learn from others and see the potential of comparisons across units.

Their ambivalence is related to the relevance of performance indicators. Unit managers do not see how quality can be measured in a simple, reliable, and valid manner. Their general approach to quality development is more qualitative and in keeping with the logic of quality cycles, and they fear that measurement by numbers will be biased, irrelevant, or of no use. They are especially skeptical about the possibility of measuring the quality of everyday life issues in long-term (often life-long) care, whereas it would be more relevant in short-term units with more clear-cut treatment goals. In keeping with this, we note fewer reservations in the more treatment-oriented units for people with mental health issues. One should, however, keep in mind that among our interviewees, only one unit manager concluded that performance measurements are unwanted since they are based on a type of logic that conflicts with the ethos of the service. Most unit managers expressed ambivalence about this point and asked for the tools that quality indicators eventually can become. A representative statement is:

*What are actually good indicators or measurements of the quality of personalized in-home services? Can some-one please provide me the book of answers to that?* (Unit manager)

There are some similarities between motivations at the local authority and service levels but in a context that is strikingly different. The meaning of performance measurement for unit managers is mainly about outcomes for users (i.e., the quality of services) rather than efficient management of the local health and care sector.

### The Choice of Measurements

#### National Level—The Need for Better Measurement of User Outcomes

The approach to the choice of measurements at the national level is dual. Municipalities are expected to report on a fixed set of variables that are plugged into the national monitoring system (mainly statistics on users, services, and expenditures). However, when it comes to performance measurements employed at the local or service levels, national documents are less specific. In keeping with the general principles in the division of labor between levels of government in Norway, the national government can decide which tasks the local authorities should deliver but not how this is performed or organized. Thus, the choice of indicators or measurements at the local or service levels is up to the local authorities, and the same applies to the extent to which local authorities use measuring as a part of their quality assurance system. One should, however, note three recommendations from the national government:

- Existing performance measurements tend to rely mostly on input and process indicators, and there is a need to develop more indicators on user outcomes.
- User surveys should be among the measurements.
- The Directorate of Health should develop a set of national quality indicators that the local authorities can chose to employ.

The national indicators proposed by the Directorate have been gradually developed and consisted of 174 indicators in 2020. The majority address specialized treatments in hospitals. Only 31 apply to local health and care. These local level indicators are heavily biased toward nursing homes, and only nine indicators are relevant for community disability services. Among these nine indicators, two address staff (% with relevant education, sick leave statistics), two are on waiting lists, and five on numbers receiving specific services. There appears to be agreement that the nationally proposed indicators for community care need improvement and that current indicators do not align with general guidelines for quality indicators (i.e., more on user outcomes). Therefore, for the next planning period, the Directorate of Health is asked to prioritize the development of indicators for this service sector.

#### Local Authority and Service Levels—the Back-Door of Administrative Systems

There appears to be two general “principles” guiding the development of indicators at the municipal level. The first is that one hardly looks to the national indicators, and unit managers were generally not even aware that these indicators existed. Sick-leave statistics and the proportion of staff with relevant education are frequently used locally and recommended nationally, but this similarity appears to be by coincidence rather than because municipalities employ national indicators. Second, the preferred indicators are those that can be automatically produced through the current administrative systems, which are primarily input and process indicators:

*It is rather homemade, and we sort of approach indicators through the back-door of our administrative and accounting systems*. (Top management)

*We are mainly using what could be generated from our existing administrative and accounting systems, and in a few cases, we count manually what cannot be generated automatically*. (Top management)

There is currently some optimism about what can be retrieved through this back-door in the near future, as this region of Norway is establishing a new comprehensive administrative IT-system for the health and care sector. This system is supposed to provide more possibilities, but so far, the extent to which this includes measurement of user outcomes remains unclear.

The two general “principles” do not tell the full story. There is choice involved in what is generated from the administrative systems, and this type of data includes “feed-back mechanisms” such as registration of complains or other types of non-conformances (e.g., accidents, deviation from expected delivery according to individual plans/statements). Some municipalities publish this type of data as part of a transparency policy, while for others, the data is part of internal quality assessments. Furthermore, both user and employee surveys are common tools among performance measurements.

The larger municipalities tend to have a more systematic and elaborate approach to performance measurement than the small municipalities where the system is more “trust-based.” The larger municipalities participating in the ASSS-collaboration tend, on a regular basis, to discuss what measurements to include in which service sector. People from different service sectors participate in working groups that outline the indicators that could be compared between municipalities. The interviewees express a reasonable level of influence on choice of ASSS-indicators, but they also argue that indicators of user outcomes are difficult to establish. The main body of indicators are thus centered on input and process variables, such as expenditures, expenditures per user, number of recipients, number of recipients with intellectual disability, proportion of staff with relevant education, and sick-leave statistics. In short, they are performance measurements strongly linked to the “management of resources” logic. Such indicators appear to be viewed as vital for top management and the municipal councils. This is because they are struggling with limited budgets and fiscal problems. Furthermore, top management appear to be fully aware that these are resource management indicators rather than indicators of quality for users. However, the indicators are still frequently referred to as quality indicators.

At the local authority level, the ASSS-indicators are used, but municipalities also tend to have a more elaborate set of indicators. There is, however, variation between service sectors, and community care appears to lag behind. One large municipality is currently working on a quality report system for services for adults with intellectual disabilities inspired by the current system in nursing homes. This system includes measurement of user satisfaction, staff formal qualifications, the proportion of part-time staff, medication, incidences of non-conformance, nutrition, and waiting lists. The intention is to measure quality of service provision, not outcomes for users, as user outcomes are believed to be too difficult to measure and strongly dependent on individual preferences. The type of measurements included in these reports are less about “management of resources” and more related to structural dimensions that may impact quality. So far, such reports are in the emergence state in services for disabled people.

Another source of information about current indicators at the municipal and/or service levels is the interviews with unit managers. When first asked about existing quality indicators, the unit managers (with a few exceptions) were rather reluctant to answer and even evasive. They felt that they should do quality measuring but had no system in operation. However, when we changed the question to what their unit has to report on and are measured by, the response tended to be: “Everything”.

*We report on sick leave, use of restraint – there is frequent auditing on use of restraint – user surveys, staff surveys every other year, economy of course. We must report on almost everything; thus, we are measured in a large number of areas*. (Unit manager)

Disregarding details, the issues that are frequently mentioned by unit managers can be grouped into four categories: (1) budget and economy, (2) human resources, (3) employer policy, and (4) compliance with procedures. Human resources are indicators such as sick-leave, part-time work, proportion with relevant education, turnover, etc. Employer policy is concerned with reducing part-time employment, the number of staff members with minority backgrounds, climate footprint, and whether the employer is viewed as attractive. Compliance with procedures includes the number of employee development interviews, checklists for completion of activities listed in the users' daily schedule, checklists of performed administrative tasks, counting instances of non-conformance, and deviations in medication handling (in brief, ticking of boxes about whether a procedure was complied with or not). However, when it boils down to what is most important, the rather uniform answer from unit managers is versions of this:

*If I should rank the ten most important things the unit is measured on, it is budget and economy from number one to ten*. (Unit manager)

This does not mean that unit managers oppose performance measurements or that they are stuck with an unsatisfactory measurement system. Their attitude to measurement is ambivalent; they want it but cannot quite grasp how to measure or quantify issues of importance. They also feel that they can influence what is measured, for instance through the dialogues with top management on strategy, aims and measurement. Some are invited into working groups proposing indicators which should be possible to retrieve from the new administrative IT-system. The main problems from the perspective of unit managers appear to be two, (i) that they cannot grasp how to measure or quantify the issues of importance (i.e., the quality of care for users) and (ii) that they hardly get any feedback on what they report to superior levels of the organization. This means that there are not many efforts to establish performance measurements at the service level and that local authority and service levels are not easily distinguished regarding choice of indicators. The local indicators tend to be chosen by the top administration of the local authority for the purpose of governance. This does not mean that unit managers have little involvement in quality assessment and development, but the activities in the service unit are based on qualitative assessments and discussions among staff, rather than quantification and performance measurement.

In brief, with respect to the choice of performance indicators, data clearly supports the hypothesis of a process bias as well as a bias toward measurements of importance for the management of resources rather than outcomes for users. So far, we conclude that there is an obvious risk of goal displacement, i.e., that measurement of quality is transformed into governance data. Thus, the national recommendation about stronger focus on measurement of outcome for users appears timely.

### The “Doing” of Measurements

Our data does not provide the opportunity to identify clear impacts of performance measurements on services, and it is likely that these types of measurements do not in themselves have dramatic effects. The point is rather how they interact with or strengthen other mechanisms. Thus, our analytical strategy was to identify clues in the interviews that are likely to impact on services together with other mechanisms, with special attention paid to unintended or potentially aversive effects. With respect to intended effects, interviewees did not talk about actual experiences but rather emphasized what they hoped for, and their hopes aligned with the motivation for wanting performance measurements. In this section, we will only address the local and service levels because the main issue at the national level is monitoring and policy rather than “doing.”

#### Local Level—Performance Measurement as a Tool in a “Race to the Bottom”?

Performance measurements at the local level primarily center on resource management, sector governance, and input/process indicators. The doing of such indicators appears to be related mainly to the monitoring of how the municipality compares with other municipalities. The logic of this type of benchmarking is to look to others to learn, and it is the political context of benchmarking, rather than the benchmarking as such, that is likely to impact services since the benchmark can be used both for increasing and decreasing ambitions. For instance, in the 1990s, Norwegian municipalities looked to Sweden and argued that “we should do at least as good as them” (25). The point was to learn from someone that presumably offered quality services. The same reasoning applies when municipalities compare the proportion of staff with relevant formal education. If the proportion is low, this is an incentive to recruit more people that have completed higher levels of education. However, according to unit managers, the main current issue is the costs. The local authorities experience fiscal strain, and there is a constant search for cost-cutting opportunities. If a sector spends more than the same sector in other municipalities, it is treated a candidate for cost-cutting initiatives. Thus, one learns from those who spend less on the sector. Several Norwegian municipalities that look for such possibilities have engaged consultancy companies that specialize in analyzing variation in expenditures across municipalities, and the result is frequently cost-cutting proposals (26).

This result is not in itself a consequence of measurements, benchmarking, or learning from others, but in the context of a search for cost-cutting, the outcome resembles what economists term “the race to the bottom.” This refers to countries or companies that cut wages, taxes, labor standards, or social security to improve their competitiveness, and those cutting most will lead the development. In the case of benchmarking of costs for community care for disabled people, municipalities learn from those who spend the least. This is not caused by performance measurements, but the measurements provide facts and arguments for cost-cutting efforts. One consequence is that the main “ten issues” that emerge in the development talks between top management and unit leaders center on budget and costs. At the dialogue conference, representatives from user organizations also claimed that “quality indicators” end up in being used as cost-cutting instruments.

As suggested above, one motivation for the use of performance measurement is the management of expectations. We see no clear cases of such “doing” in our data, but the phenomenon can be observed in local newspapers when they publish criticisms from users, family, or disability organizations that tend to be illustrated by the situation of a specific individual. A typical response from the local authorities is that they cannot comment on the specific case; instead, they refer to statistics showing that they spend as much money as other municipalities or they point to user surveys that show reasonable levels of satisfaction. Thus, the performance measurements are not directly used to manage expectations but rather to defend the current level and quality of services against criticisms.

These results point to adverse effects of performance measurement. This does not mean that local authorities only use performance measurement for “bad” purposes. Measurements are also used for identification of problems or service units that need to improve and above all monitoring for the purpose of planning and resource management within the sector. However, this monitoring goes on in the central administration and primarily affects service delivery in the form of budget decisions. According to unit managers who attended the dialogue conference, there is limited communication across organizational levels about performance measurement results.

#### Service Level—The Impact of a Process Bias

The issues that unit managers report on, excepting budget, staff, and costs, concerns compliance with procedures (i.e., ticking boxes to show whether a task is done or not). The logic of this as part of quality assurance, is that it safe-guards that expected activities or tasks are performed. These tasks could be related to resident activities or different types of staff documentation, such as completing an annual review of a user service plan, organizing meetings with families, reporting instances of non-compliance, etc. The documentation of performed resident activities is important in services that involve a number of part-time staff and extensive use of substitutes. Parts of the reporting is supposed to function as milestones where one reflects on how things are going and possible needs for change. This is, for example, the purpose of the annual review of the user service plan. Ticking procedural boxes is thus unlikely to have any adverse impacts on services because this process is meant to ensure that certain activities are completed. None of the interviewees were skeptical about this, but they did recognize that checking whether a report was delivered or not was an incomplete method of quality assurance. They saw the need to address the content of the report to assess whether it was really used as a milestone. Presently, this did not usually occur unless unit managers received other types of information that suggested a need for action.

Our critical analysis at this point is based on focus group interviews with direct care staff from the earlier study that examined extensive services in other peoples' home. When describing their work, the direct care staff present it as predetermined by the daily schedule for each user and that they have established a set of routines and must-do-tasks that ensures that expected tasks are done. When asked about the reason behind these routines, the typical answer was a variation of the following statement:

*I do not know. They were here before I started to work here. I do not know who has written them. But we need them. There is a lot of people working here, and if we do not follow strict routines, things may be forgotten*. (19, p. 170)

The professional discretion and reasoning that this type of documentation is meant to ensure, slips into routinization and an unreflective performance of required tasks. This is similar to the criticism of performance measurement raised by those who point to the risk of “indicators replacing goals”, with professional discretion and individual tailoring losing ground. Some informants suggested that they are trapped in certain ways of doing things: “*and we have done so for 20 years”* (19, p. 170). The intention of some of the process measurements, such as ticking off that “review of individual plan is finalized,” is intended to counteract the possibility of being caught in these types of traps. However, the totality of ticking off boxes runs the risk of reinforcing this routinization trap, because what is measured is what's scheduled. The documentation is just another administrative task to be completed, more than a milestone, and most unit managers were fully aware of this risk.

There are, of course, some reservations to this rather depressing image. First, there is variation across services with respect to whether the “milestone procedures” are simply routine (19). Some services use such milestones to actively to reflect on the current service. Second, there is a distinction between short-term services for people with mental health issues and long-term services for people with intellectual disabilities. To a large extent, the unit managers involved in mental health services did use the milestone procedures as intended; that is, for goalsetting, evaluation, reflection, and, if needed, for change.

## Discussion

This article has addressed i) the motivation behind the introduction of performance measurement in community services for disabled people, ii) the choice of indicators in actual use, and iii) the possible impact on services. In the empirical context of Norway, indicators of outcome quality for users are a subset of a wider movement toward performance measurement, and one question has been to what extent measurement of service quality slips into the measurement of service production factors.

The findings show that one can identify multiple motivations for the introduction of performance measurement. At the national level, policy documents explicitly refer to the need for a facts-based and transparent governance, tools for quality assurance, and to provide local and national authorities information needed for the management and planning of the sector (24, p. 22). Measurements are expected to be sensitive to user needs and quality outcome for users. To improve the quality of outcomes for users is also a motivation at the local authority level, but this appears to be overshadowed by needs concerning the management of the sector. At the service unit level, the motivation for performance measurements is foremost as a tool for the development of service quality. Unit managers welcome more use of performance measurements, but they cannot really grasp how the important issues (i.e., quality outcomes for users) can be measured or quantified. Their attitude can be summarized as ambivalent, including a call for better measurement of outcome quality for users.

In practice, local authorities chose which measurements are in operation at the service level. The national government offers some guidance, but in the context of community care, even guidance is underdeveloped. The national authorities do, however, require that local authorities report on a set of measurements relevant to national monitoring and planning. Regarding community services for disabled people, this reporting is basically statistics on service provision (i.e., the number of users and expenditures), whereas for other parts of the health and care system, the reporting also comprises quality indicators, such as regularity of medical checks in nursing homes. At the local authority level, the main driver of choice of measurements is practical—the measurements are what can be retrieved from administrative systems. These measurements are mainly input and process factors, and the most attended indicators appear to be related to expenditures, followed by other information of relevance for the management of the sector. Service units also report on a set of process factors, some of which have potential utility for service quality. This includes reporting on milestones, such as annual reviews of individual service plans, and non-conformance. This reporting is potential tools to uncover shortcomings that need to be acted upon. Many local authorities also conduct user surveys.

As for the “doing” of performance measurements, our data suggests a clear distinction between “up-stream” and “down-stream” information. The up-stream model means that lower levels are providing governance information to higher levels (from local authorities to the national level and from service units to the municipal administration). This information is used for planning, budgeting, management of the sector, and for reporting to political bodies. In community care for disabled people, less information appears to be going “down-stream” to the practical delivery of services. The role of performance measurements in the development of service quality appears to be limited. We do, however, see some indirect impact that is partly related to the local authorities' use of expenditure data and partly related to practices of documentation. The local authorities operate in a context with fiscal strain and have strong incentives to look for cost-cutting strategies. Thus, when comparing themselves with other municipalities, local authorities search for sectors where they spend more than average to identify candidates for cost-cutting. This mechanism resembles the so-called “race to the bottom” and is, at the service level, likely to be experienced as budget cuts. We have also identified a mechanism where routinised reporting on process indicators may turn out to be, not a milestone for evaluation and reflection, but rather part of a general routinization of the service at the expense of professional discretion and individual tailoring. However, this finding is uncertain and may easily be counteracted by a more active practice in the use of such reporting.

The most common user-oriented quality tool is user surveys where reasonable levels of satisfaction tend to be used by local authorities as defense against criticisms. There is, however, reason to ask whether this is a valid and reliable measure. Unit managers comment that they do not trust these measurements because users frequently do not respond independently, many respondents are reluctant to criticize services that they are dependent on, and response rates are very low. This comes in addition to the general homeostatic effects on responses to satisfaction surveys (27), that is, one adapts expectations to the factual situation. The consequence is that, in general, such surveys end up with satisfactions rates of 70–80%, irrespective of the actual situation. Measurement of user satisfaction by surveys thus runs the risk of being insensitive to the quality of care (and that is maybe why they are so popular?).

This study set out to analyze how performance measurement was used in the quality assurance of community services for disabled people. It was inspired in part by studies of the Norwegian Work and Welfare Administration that show there is no correlation between doing well on service quality indicators and success regarding the real goal—to support people into employment (28). This appeared to be a typical case of “indicators replacing goals” due to the strategic behavior of staff. We thus searched for clues about whether the points referred to in the critical literature on performance measurement were in operation and whether the intended benefits were present. We can clearly identify motivations related to transparency, accountability, and benchmarking, and these types of mechanisms also appear to be present in the “up-stream” part of the “doing.” As such, this resembles another key point in New Public Management: managerialism. From the point of view of the national government, the performance measurement provides hard facts input to the monitoring and planning of the sector, and for the local authorities, the data is useful tools in budgeting and planning processes. From the perspective of users, however, this is hardly beneficial.

As for the main critical points, such as tunnel vision or target fixation, we have observed few signs of strategic behavior from staff that may lead to this. This may be because the art of performance measurement is underdeveloped in community services for disabled people in Norway and is generally not used at the staff-member level. Our lack of findings should thus not be seen as evidence for the non-existence of such mechanisms. What is evident in our data, however, is the process bias—that one uses indicators that are easily retrievable from administrative systems and that such indicators tend to be input and process factors rather than outcomes for users.

One should note (i) that there appears to be differences depending on the size of the municipality, as smaller municipalities measure less and operate more qualitatively, and (ii) that some of the dubious effects appear to be more evident in long-term services for people with intellectual disabilities, and that the employment of outcome indicators are more used and more useful in short-term services for people with mental health problems. Furthermore, it is essential to highlight that the findings of this study hardly are effects of performance measurement per se, but rather dependent on the context, practical use, and orientation of the indicators in use. The dominance of production indicators does something to the “doing” of indicators. In keeping with this, unit managers tend to expect clear benefits if the measurement of outcome quality for users is introduced. This is among others because such measurements can be used by unit managers to balance the current focus on budget issues in their annual reviews with top management, and possibly also have an impact in budget discussions in the municipal council. They also see potential use in internal quality development efforts.

Our preliminary conclusion is not that one should refrain from performance measurement, but that there is an urgent need to help unit managers with the tools they need to introduce better and more useful indicators of outcomes for users. We do, however, also want to issue a warning that in real-life contexts where quality development meets the economic worries of local authorities, a transformation of well-intended measurements is not unlikely. As a managerial tool, the impact of performance measurements is likely to be context dependent and ambiguous. It is tempting to argue the case of a more qualitative approach to quality development based on user involvement and co-production, professional discretion, and managerial leadership. In principle, this will be a return to the use of Deming's cycle (16). However, performance measurements appear to have gained an irreversible position in the management of health and care services, and the dominance of measurements related to governance information needs to be balanced by more user-oriented measurements. However, at the service level, this should be a supplement rather than a replacement of professional discretion and individual tailoring, and employed as a part of a qualitatively oriented quality cycle.

Finally, one should note a set of reservations about the results presented in this article. First, our study is based on a limited set of interviews with informants, and the narratives from people in the same position were unusually varied. Second, our data primarily comes from documents and interviews at the management level, not hands-on staff or users/user representatives. The missing user perspective is a clear limitation. It is, however, not likely that users or their family have much insight into the current use of performance measurement, but representatives from user associations could add to the current data like they did during the dialogue conference. First-line staff could also contribute to perspectives on their reporting, and to what extent they experience that the reporting affects their doing or is fed back as part of the efforts to identify areas in need of development. To include user and first-line staff perspectives would be a task for future research. Lastly, this study was conducted in a system that is still in the making, as the use of performance measurements are underdeveloped in community care for disabled people. This means (i) that uncovering unintended consequences due to strategic behavior of staff or unit management was less likely than in a more established and elaborated system and (ii) that existing measurements are dominated by administrative indicators that can potentially be applied in any sector, whereas sector-specific measurements play a minor role or no role at all. Thus, the potential of user-oriented measurements to balance the impact of the dominant production-oriented measures has not been studied.

## Data Availability Statement

The original contributions presented in the study are included in the article/supplementary material, further inquiries can be directed to the corresponding author.

## Author Contributions

JT has been lead author and there are equal contributions to data gathering and analyses. All authors have contributed to the data gathering, analysis, and writing of the manuscript. All authors contributed to the article and approved the submitted version.

## Funding

Funded by the Research Council of Norway (grant number 302878/H40), only role is as funding agency.

## Conflict of Interest

The authors declare that the research was conducted in the absence of any commercial or financial relationships that could be construed as a potential conflict of interest.

## Publisher's Note

All claims expressed in this article are solely those of the authors and do not necessarily represent those of their affiliated organizations, or those of the publisher, the editors and the reviewers. Any product that may be evaluated in this article, or claim that may be made by its manufacturer, is not guaranteed or endorsed by the publisher.
